# Diaqua­bis(4-chloro­benzoato-κ*O*)bis­(*N*,*N*-diethyl­nicotinamide-κ*N* ^1^)manganese(II)

**DOI:** 10.1107/S1600536808005540

**Published:** 2008-02-29

**Authors:** Tuncer Hökelek, Nagihan Çaylak, Hacali Necefoğlu

**Affiliations:** a Department of Physics, Hacettepe University, 06800 Beytepe, Ankara, Turkey; b Department of Physics, Faculty of Arts and Sciences, Sakarya University, 54187 Esentepe, Adapazarı, Turkey; c Department of Chemistry, Kafkas University, 63100 Kars, Turkey

## Abstract

The title compound, [Mn(C_7_H_4_ClO_2_)_2_(C_10_H_14_N_2_O)_2_(H_2_O)_2_], is a monomeric complex with the Mn^II^ atom lying on an inversion center. It contains two 4-chloro­benzoate and two diethyl­nicotinamide ligands and two water mol­ecules, all of which are monodentate. The four O atoms in the equatorial plane around the Mn atom form a slightly distorted square-planar arrangement, while the distorted octa­hedral geometry is completed by two N atoms in the axial positions. In the crystal structure, O—H⋯O hydrogen bonds link the mol­ecules into an infinite chain.

## Related literature

For general background, see: Adiwidjaja *et al.* (1978 ▶); Amiraslanov *et al.* (1979 ▶); Antolini *et al.* (1982 ▶); Antsyshkina *et al.* (1980 ▶); Nadzhafov *et al.* (1981 ▶); Shnulin *et al.* (1981 ▶). For related structures, see: Hökelek *et al.* (1995 ▶, 1997 ▶); Hökelek *et al.* (2007 ▶); Hökelek & Necefoğlu (1996 ▶, 1997 ▶, 2007 ▶).
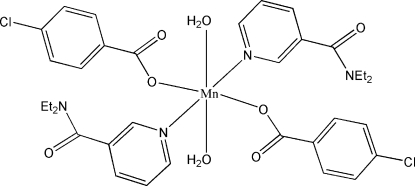


## Experimental

### 

#### Crystal data

[Mn(C_7_H_4_ClO_2_)_2_(C_10_H_14_N_2_O)_2_(H_2_O)_2_]
*M* *_r_* = 758.54
Triclinic, 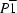

*a* = 7.3552 (1) Å
*b* = 8.6465 (2) Å
*c* = 15.9847 (3) Å
α = 84.500 (16)°
β = 78.616 (17)°
γ = 68.154 (17)°
*V* = 924.73 (12) Å^3^
*Z* = 1
Mo *K*α radiation
μ = 0.56 mm^−1^
*T* = 294 (2) K
0.30 × 0.15 × 0.10 mm

#### Data collection

Enraf–Nonius TurboCAD-4 diffractometer
Absorption correction: ψ scan (North *et al.*, 1968 ▶) *T*_min_ = 0.902, *T*_max_ = 0.950
4010 measured reflections
3752 independent reflections
2604 reflections with *I* > 2σ(*I*)
*R*_int_ = 0.062
3 standard reflections frequency: 120 min intensity decay: 1%

#### Refinement

*R*[*F* ^2^ > 2σ(*F* ^2^)] = 0.080
*wR*(*F* ^2^) = 0.254
*S* = 1.04
3752 reflections
225 parameters
5 restraints
H atoms treated by a mixture of independent and constrained refinement
Δρ_max_ = 1.26 e Å^−3^
Δρ_min_ = −1.31 e Å^−3^

### 

Data collection: *CAD-4 EXPRESS* (Enraf–Nonius, 1989 ▶); cell refinement: *CAD-4 EXPRESS*; data reduction: *XCAD4* (Harms & Wocadlo, 1995 ▶); program(s) used to solve structure: *SIR92* (Altomare *et al.*, 1994 ▶); program(s) used to refine structure: *SHELXL97* (Sheldrick, 2008 ▶); molecular graphics: *ORTEP-3* (Farrugia, 1997 ▶); software used to prepare material for publication: *WinGX* (Farrugia, 1999 ▶).

## Supplementary Material

Crystal structure: contains datablocks I, global. DOI: 10.1107/S1600536808005540/hy2120sup1.cif

Structure factors: contains datablocks I. DOI: 10.1107/S1600536808005540/hy2120Isup2.hkl

Additional supplementary materials:  crystallographic information; 3D view; checkCIF report

## Figures and Tables

**Table:** 

Mn—O1	2.141 (3)
Mn—O4	2.205 (4)
Mn—N1	2.281 (4)

**Table:** 

O1^i^—Mn—O4	90.38 (14)
O1—Mn—O4	89.62 (14)
O1—Mn—N1^i^	92.23 (14)
O4—Mn—N1^i^	92.72 (14)
O1—Mn—N1	87.77 (14)
O4—Mn—N1	87.28 (14)

Symmetry code: (i) 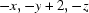.

**Table 2:** Hydrogen-bond geometry (Å, °)

*D*—H⋯*A*	*D*—H	H⋯*A*	*D*⋯*A*	*D*—H⋯*A*
O4—H41⋯O2^i^	0.99 (4)	1.71 (5)	2.670 (6)	162 (7)
O4—H42⋯O3^ii^	0.93 (5)	1.85 (5)	2.766 (6)	168 (7)

Symmetry codes: (i) 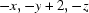; (ii) 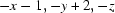.

**Fig. 1.**
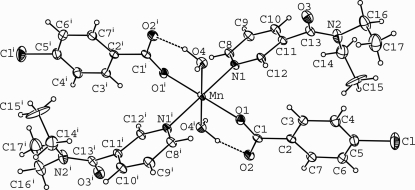
The molecular structure of the title compound. Displacement ellipsoids are drawn at the 20% probability level. Hydrogen bonds are shown as dashed lines. [Symmetry code: (i) -x, 2 - y, -z.]

**Fig. 2.**
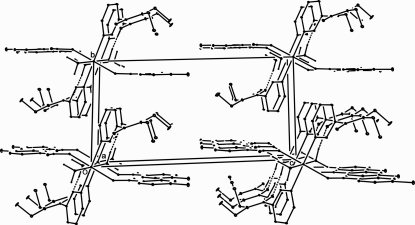
A packing diagram of the title compound, viewed down the a-axis, showing hydrogen bonds (dashed lines) linking the molecules into chains. H atoms not involved in hydrogen bonds have been omitted for clarity.

## supplementary crystallographic information

### Comment

Transition metal complexes with biochemical molecules show interesting physical and/or chemical properties, through which they may find applications in biological systems (Antolini *et al.*, 1982). The structural functions and coordination relationships of the arylcarboxylate ions in manganese(II) complexes of benzoic acid derivatives may be changed, depending on the nature and position of the substituted groups on the benzene ring, the nature of the additional ligand molecule or solvent, and the medium of the synthesis (Adiwidjaja *et al.*, 1978; Amiraslanov *et al.*, 1979; Antsyshkina *et al.*, 1980; Nadzhafov *et al.*, 1981; Shnulin *et al.*, 1981).

*N*,*N*-Diethylnicotinamide (DENA) is an important respiratory stimulant. The structures of several complexes obtained by reacting divalent transition metal ions with DENA have been determined in our laboratory, including those of Cu_2_(DENA)_2_(C_6_H_5_COO)_4_, (II), (Hökelek *et al.*, 1995), [Zn_2_(DENA)_2_(C_7_H_5_O_3_)_4_].2H_2_O, (III), (Hökelek & Necefoğlu, 1996), [Co(DENA)_2_(C_7_H_5_O_3_)_2_(H_2_O)_2_], (IV), (Hökelek & Necefoğlu, 1997), [Cu(DENA)_2_(C_7_H_4_NO_4_)_2_(H_2_O)_2_], (V), (Hökelek *et al.*, 1997) and [Zn(DENA)_2_(C_7_H_4_FO_2_)_2_(H_2_O)_2_], (VI), (Hökelek *et al.*, 2007). The structure determination of the title compound, (I), a manganese(II) complex with two chlorobenzoate (CB), two DENA ligands and two water molecules, was undertaken in order to determine the properties of the CB and DENA ligands and also to compare the results obtained with those reported previously.

Compound (I) is a monomeric complex, with the Mn atom lying on a center of symmetry. It contains two CB, two DENA ligands and two water molecules (Fig. 1). All ligands are monodentate. The four O atoms (O1, O4, and their symmetry-related atoms) in the equatorial plane around the Mn atom form a slightly distorted square-planar arrangement, while the slightly distorted octahedral coordination geometry is completed by the two N atoms of the DENA ligands in the axial positions (Table 1 and Fig. 1).

The near equality of the C1—O1 [1.256 (6) Å] and C1—O2 [1.245 (6) Å] bonds in the carboxylate group indicates a delocalized bonding arrangement, rather than localized single and double bonds, and may be compared with the corresponding distances: 1.259 (9) and 1.273 (9) Å in (II), 1.279 (4) and 1.246 (4) Å in (III), 1.251 (6) and 1.254 (7) Å in (IV), 1.278 (3) and 1.246 (3) Å in (V) and 1.265 (6) and 1.275 (6) Å in [Mn(C_9_H_10_NO_2_)_2_(H_2_O)_4_].2H_2_O, (VII), (Hökelek & Necefoğlu, 2007). This may be due to the intramolecular O—H···O hydrogen bond involving the carboxylate O atom (Table 2). In (I), the average Mn—O bond length is 2.173 (3) Å. The Mn atom is displaced out of the least-squares plane of the carboxylate group (O1/C1/O2) by 0.890 (1) Å; this is reported as 2.185 (4) and 1.365 (3) Å, respectively, in (VII). The dihedral angle between the planar carboxylate group and the benzene ring A (C2 to C7) is 3.0 (4)°, while that between rings A and B (N1/C8 to C12) is 81.0 (4)°.

As can be seen from the packing diagram (Fig. 2), the Mn atoms are located at the corners of the unit cell and the molecules of (I) are linked into infinite chains along the *a*-axis by intermolecular O—H···O hydrogen bonds (Table 2).

### Experimental

The title compound was prepared by the reaction of Mn(NO_3_)_2_ (1.79 g, 10 mmol) in H_2_O (25 ml) and DENA (3.56 g, 20 mmol) in H_2_O (25 ml) with sodium *p*-chlorobenzoate (3.57 g, 20 mmol) in H_2_O (100 ml). The mixture was filtered and set aside to crystallize at ambient temperature for several days, giving colorless single crystals.

### Refinement

H atoms of the water molecule were located in a difference Fourier map and refined isotropically. The remaining H atoms were positioned geometrically and refined as riding atoms, with C—H = 0.93 (aromatic), 0.97 (methylene) and 0.96 Å (methyl) and with *U*_iso_(H) = *xU*_eq_(C), where *x* = 1.0 for H atoms of C15 methyl, 1.5 for H atoms of C17 methyl, and 1.2 for other H atoms. The restrains on the C14—C15 bond length and O—H bond lengths and H—O—H bond angle of water molecule were applied. The highest residual electron density was found 0.92 Å from H15B and the deepest hole 0.14 Å from C15.

### Crystal data

**Table:**

[Mn(C_7_H_4_ClO_2_)_2_(C_10_H_14_N_2_O)_2_(H_2_O)_2_]	*Z* = 1
*M**_r_* = 758.54	*F*_000_ = 395
Triclinic, *P*1	*D*_x_ = 1.362 Mg m^−^^3^
Hall symbol: -P 1	Mo *K*α radiation λ = 0.71073 Å
*a* = 7.3552 (1) Å	Cell parameters from 25 reflections
*b* = 8.6465 (2) Å	θ = 5.2–11.6º
*c* = 15.9847 (3) Å	µ = 0.56 mm^−^^1^
α = 84.500 (16)º	*T* = 294 (2) K
β = 78.616 (17)º	Block, colorless
γ = 68.154 (17)º	0.30 × 0.15 × 0.10 mm
*V* = 924.73 (12) Å^3^	

### Data collection

**Table:**

Enraf–Nonius TurboCAD-4 diffractometer	*R*_int_ = 0.062
Radiation source: fine-focus sealed tube	θ_max_ = 26.3º
Monochromator: graphite	θ_min_ = 3.0º
*T* = 294(2) K	*h* = −8→9
ω scans	*k* = 0→10
Absorption correction: ψ scan(North *et al.*, 1968)	*l* = −19→19
*T*_min_ = 0.902, *T*_max_ = 0.950	3 standard reflections
4010 measured reflections	every 120 min
3752 independent reflections	intensity decay: 1%
2604 reflections with *I* > 2σ(*I*)	

### Refinement

**Table:**

Refinement on *F*^2^	Secondary atom site location: difference Fourier map
Least-squares matrix: full	Hydrogen site location: inferred from neighbouring sites
*R*[*F*^2^ > 2σ(*F*^2^)] = 0.080	H atoms treated by a mixture of independent and constrained refinement
*wR*(*F*^2^) = 0.254	*w* = 1/[σ^2^(*F*_o_^2^) + (0.1471*P*)^2^ + 1.5562*P*] where *P* = (*F*_o_^2^ + 2*F*_c_^2^)/3
*S* = 1.04	(Δ/σ)_max_ < 0.001
3752 reflections	Δρ_max_ = 1.26 e Å^−^^3^
225 parameters	Δρ_min_ = −1.31 e Å^−^^3^
5 restraints	Extinction correction: none
Primary atom site location: structure-invariant direct methods	

### Fractional atomic coordinates and isotropic or equivalent isotropic displacement parameters (Å^2^)

**Table:**

	*x*	*y*	*z*	*U*_iso_*/*U*_eq_	
Mn	0.0000	1.0000	0.0000	0.0323 (3)	
Cl	−0.7397 (3)	0.8445 (3)	0.46709 (13)	0.0925 (7)	
O1	−0.1119 (5)	0.8758 (4)	0.1086 (2)	0.0408 (8)	
O2	0.0744 (5)	0.8690 (5)	0.2036 (3)	0.0508 (10)	
O3	−0.8388 (6)	1.3270 (5)	0.1267 (3)	0.0576 (11)	
O4	−0.2247 (6)	0.9829 (5)	−0.0681 (2)	0.0463 (9)	
H41	−0.195 (10)	1.042 (7)	−0.122 (2)	0.07 (2)*	
H42	−0.221 (12)	0.880 (5)	−0.083 (4)	0.08 (2)*	
N1	−0.2326 (6)	1.2447 (5)	0.0532 (3)	0.0360 (9)	
N2	−0.8349 (8)	1.4183 (8)	0.2506 (4)	0.0649 (15)	
C1	−0.0808 (7)	0.8688 (6)	0.1836 (3)	0.0366 (11)	
C2	−0.2462 (7)	0.8636 (5)	0.2547 (3)	0.0350 (10)	
C3	−0.4206 (7)	0.8580 (6)	0.2372 (3)	0.0381 (11)	
H3	−0.4358	0.8589	0.1807	0.046*	
C4	−0.5733 (8)	0.8509 (7)	0.3024 (4)	0.0487 (13)	
H4	−0.6898	0.8463	0.2904	0.058*	
C5	−0.5477 (9)	0.8509 (8)	0.3850 (4)	0.0525 (14)	
C6	−0.3789 (9)	0.8586 (8)	0.4047 (4)	0.0533 (14)	
H6	−0.3655	0.8585	0.4613	0.064*	
C7	−0.2282 (8)	0.8665 (7)	0.3392 (3)	0.0434 (12)	
H7	−0.1138	0.8739	0.3520	0.052*	
C8	−0.2050 (7)	1.3893 (6)	0.0424 (3)	0.0390 (11)	
H8	−0.0855	1.3917	0.0107	0.047*	
C9	−0.3452 (8)	1.5356 (6)	0.0760 (4)	0.0461 (13)	
H9	−0.3203	1.6343	0.0676	0.055*	
C10	−0.5227 (8)	1.5332 (6)	0.1223 (4)	0.0442 (13)	
H10	−0.6197	1.6304	0.1458	0.053*	
C11	−0.5554 (7)	1.3848 (6)	0.1334 (3)	0.0359 (11)	
C12	−0.4080 (7)	1.2446 (6)	0.0965 (3)	0.0380 (11)	
H12	−0.4315	1.1453	0.1020	0.046*	
C13	−0.7519 (7)	1.3715 (6)	0.1710 (3)	0.0413 (12)	
C14	−0.7427 (13)	1.4762 (10)	0.3087 (5)	0.080 (2)	
H14A	−0.6247	1.4935	0.2770	0.096*	
H14B	−0.8350	1.5823	0.3318	0.096*	
C15	−0.6883 (17)	1.3572 (14)	0.3785 (7)	0.132	
H15A	−0.6290	1.3989	0.4151	0.132*	
H15B	−0.5949	1.2527	0.3558	0.132*	
H15C	−0.8052	1.3412	0.4106	0.132*	
C16	−1.0402 (11)	1.4197 (10)	0.2815 (6)	0.080 (2)	
H16A	−1.1031	1.4942	0.3291	0.096*	
H16B	−1.1177	1.4616	0.2362	0.096*	
C17	−1.0391 (16)	1.2541 (11)	0.3085 (7)	0.107 (3)	
H17A	−1.1734	1.2589	0.3272	0.160*	
H17B	−0.9659	1.2137	0.3546	0.160*	
H17C	−0.9773	1.1802	0.2614	0.160*	

### Atomic displacement parameters (Å^2^)

**Table:**

	*U*^11^	*U*^22^	*U*^33^	*U*^12^	*U*^13^	*U*^23^
Mn	0.0261 (5)	0.0275 (5)	0.0373 (6)	−0.0030 (4)	−0.0004 (4)	−0.0107 (4)
Cl	0.0696 (12)	0.144 (2)	0.0638 (12)	−0.0517 (13)	0.0190 (9)	−0.0096 (12)
O1	0.043 (2)	0.0384 (19)	0.0390 (19)	−0.0133 (16)	−0.0033 (15)	−0.0072 (14)
O2	0.034 (2)	0.061 (3)	0.057 (2)	−0.0162 (18)	−0.0097 (17)	−0.0010 (19)
O3	0.043 (2)	0.066 (3)	0.070 (3)	−0.022 (2)	−0.0078 (19)	−0.022 (2)
O4	0.043 (2)	0.046 (2)	0.054 (2)	−0.0195 (17)	−0.0090 (17)	−0.0081 (18)
N1	0.030 (2)	0.026 (2)	0.046 (2)	−0.0054 (16)	−0.0002 (17)	−0.0086 (16)
N2	0.052 (3)	0.079 (4)	0.061 (3)	−0.026 (3)	0.011 (2)	−0.024 (3)
C1	0.031 (2)	0.022 (2)	0.049 (3)	−0.0010 (18)	−0.007 (2)	−0.0055 (19)
C2	0.034 (2)	0.022 (2)	0.044 (3)	−0.0032 (18)	−0.006 (2)	−0.0050 (18)
C3	0.037 (3)	0.033 (3)	0.043 (3)	−0.012 (2)	−0.007 (2)	−0.005 (2)
C4	0.036 (3)	0.058 (3)	0.055 (3)	−0.021 (3)	−0.005 (2)	−0.005 (3)
C5	0.044 (3)	0.055 (3)	0.051 (3)	−0.015 (3)	0.005 (3)	−0.005 (3)
C6	0.055 (3)	0.064 (4)	0.038 (3)	−0.018 (3)	−0.006 (2)	−0.001 (3)
C7	0.036 (3)	0.045 (3)	0.047 (3)	−0.010 (2)	−0.008 (2)	−0.008 (2)
C8	0.032 (2)	0.032 (2)	0.051 (3)	−0.009 (2)	−0.005 (2)	−0.009 (2)
C9	0.042 (3)	0.030 (3)	0.066 (4)	−0.012 (2)	−0.007 (3)	−0.009 (2)
C10	0.040 (3)	0.027 (2)	0.059 (3)	−0.003 (2)	−0.005 (2)	−0.018 (2)
C11	0.031 (2)	0.032 (2)	0.040 (3)	−0.0040 (19)	−0.0066 (19)	−0.0103 (19)
C12	0.030 (2)	0.028 (2)	0.050 (3)	−0.0054 (19)	0.000 (2)	−0.010 (2)
C13	0.031 (2)	0.038 (3)	0.051 (3)	−0.007 (2)	−0.003 (2)	−0.013 (2)
C14	0.085 (5)	0.078 (5)	0.072 (5)	−0.029 (4)	−0.002 (4)	−0.009 (4)
C15	0.180	0.156	0.138	−0.130	−0.127	0.126
C16	0.060 (4)	0.067 (5)	0.098 (6)	−0.015 (4)	0.018 (4)	−0.026 (4)
C17	0.116 (8)	0.071 (6)	0.112 (7)	−0.031 (5)	0.030 (6)	−0.014 (5)

### Geometric parameters (Å, °)

**Table:**

Mn—O1^i^	2.141 (3)	C7—C6	1.383 (8)
Mn—O1	2.141 (3)	C7—H7	0.9300
Mn—O4	2.205 (4)	C8—H8	0.9300
Mn—O4^i^	2.205 (4)	C9—C8	1.376 (7)
Mn—N1^i^	2.281 (4)	C9—H9	0.9300
Mn—N1	2.281 (4)	C10—C9	1.373 (8)
Cl—C5	1.741 (6)	C10—H10	0.9300
O1—C1	1.256 (6)	C11—C12	1.378 (6)
O2—C1	1.245 (6)	C11—C10	1.380 (7)
O3—C13	1.214 (6)	C12—H12	0.9300
O4—H41	0.99 (4)	C13—N2	1.328 (7)
O4—H42	0.93 (5)	C13—C11	1.494 (7)
N1—C8	1.330 (6)	C14—C15	1.453 (9)
N1—C12	1.339 (6)	C14—H14A	0.9700
N2—C14	1.471 (10)	C14—H14B	0.9700
N2—C16	1.489 (9)	C15—H15A	0.9600
C2—C3	1.384 (7)	C15—H15B	0.9600
C2—C7	1.387 (7)	C15—H15C	0.9600
C2—C1	1.502 (7)	C16—C17	1.453 (11)
C3—H3	0.9300	C16—H16A	0.9700
C4—C5	1.369 (8)	C16—H16B	0.9700
C4—C3	1.388 (7)	C17—H17A	0.9600
C4—H4	0.9300	C17—H17B	0.9600
C5—C6	1.366 (9)	C17—H17C	0.9600
C6—H6	0.9300		
			
O1^i^—Mn—O1	180.000 (1)	C6—C7—H7	119.7
O1^i^—Mn—O4	90.38 (14)	C2—C7—H7	119.7
O1—Mn—O4	89.62 (14)	N1—C8—C9	122.9 (5)
O1^i^—Mn—O4^i^	89.62 (14)	N1—C8—H8	118.5
O1—Mn—O4^i^	90.38 (14)	C9—C8—H8	118.5
O4—Mn—O4^i^	180.00 (16)	C10—C9—C8	118.8 (5)
O1^i^—Mn—N1^i^	87.77 (14)	C10—C9—H9	120.6
O1—Mn—N1^i^	92.23 (14)	C8—C9—H9	120.6
O4—Mn—N1^i^	92.72 (14)	C9—C10—C11	119.2 (4)
O4^i^—Mn—N1^i^	87.28 (14)	C9—C10—H10	120.4
O1^i^—Mn—N1	92.23 (14)	C11—C10—H10	120.4
O1—Mn—N1	87.77 (14)	C12—C11—C10	118.2 (5)
O4—Mn—N1	87.28 (14)	C12—C11—C13	117.2 (4)
O4^i^—Mn—N1	92.72 (14)	C10—C11—C13	123.8 (4)
N1^i^—Mn—N1	180.0	N1—C12—C11	123.0 (5)
C1—O1—Mn	127.5 (3)	N1—C12—H12	118.5
Mn—O4—H41	100 (4)	C11—C12—H12	118.5
Mn—O4—H42	121 (5)	O3—C13—N2	120.8 (5)
H41—O4—H42	107 (4)	O3—C13—C11	119.4 (5)
C8—N1—C12	117.7 (4)	N2—C13—C11	119.7 (5)
C8—N1—Mn	123.5 (3)	C15—C14—N2	111.7 (7)
C12—N1—Mn	118.8 (3)	C15—C14—H14A	109.3
C13—N2—C14	124.8 (6)	N2—C14—H14A	109.3
C13—N2—C16	117.3 (6)	C15—C14—H14B	109.3
C14—N2—C16	117.8 (6)	N2—C14—H14B	109.3
O2—C1—O1	125.2 (5)	H14A—C14—H14B	107.9
O2—C1—C2	117.6 (5)	C14—C15—H15A	109.5
O1—C1—C2	117.1 (4)	C14—C15—H15B	109.5
C3—C2—C7	118.6 (5)	H15A—C15—H15B	109.5
C3—C2—C1	120.8 (5)	C14—C15—H15C	109.5
C7—C2—C1	120.6 (5)	H15A—C15—H15C	109.5
C2—C3—C4	121.2 (5)	H15B—C15—H15C	109.5
C2—C3—H3	119.4	C17—C16—N2	111.5 (7)
C4—C3—H3	119.4	C17—C16—H16A	109.3
C5—C4—C3	118.2 (5)	N2—C16—H16A	109.3
C5—C4—H4	120.9	C17—C16—H16B	109.3
C3—C4—H4	120.9	N2—C16—H16B	109.3
C6—C5—C4	122.2 (5)	H16A—C16—H16B	108.0
C6—C5—Cl	119.3 (5)	C16—C17—H17A	109.5
C4—C5—Cl	118.5 (5)	C16—C17—H17B	109.5
C5—C6—C7	119.1 (5)	H17A—C17—H17B	109.5
C5—C6—H6	120.5	C16—C17—H17C	109.5
C7—C6—H6	120.5	H17A—C17—H17C	109.5
C6—C7—C2	120.6 (5)	H17B—C17—H17C	109.5
			
O4—Mn—O1—C1	−164.0 (4)	C7—C2—C1—O2	2.8 (7)
O4^i^—Mn—O1—C1	16.0 (4)	C3—C2—C1—O1	3.3 (6)
N1^i^—Mn—O1—C1	103.3 (4)	C7—C2—C1—O1	−175.9 (4)
N1—Mn—O1—C1	−76.7 (4)	C7—C2—C3—C4	−1.7 (7)
O1^i^—Mn—N1—C8	−32.0 (4)	C1—C2—C3—C4	179.1 (5)
O1—Mn—N1—C8	148.0 (4)	C5—C4—C3—C2	0.5 (8)
O4—Mn—N1—C8	−122.3 (4)	C3—C4—C5—C6	0.4 (9)
O4^i^—Mn—N1—C8	57.7 (4)	C3—C4—C5—Cl	179.2 (4)
O1^i^—Mn—N1—C12	146.8 (4)	C4—C5—C6—C7	−0.1 (10)
O1—Mn—N1—C12	−33.2 (4)	Cl—C5—C6—C7	−178.9 (5)
O4—Mn—N1—C12	56.6 (4)	C2—C7—C6—C5	−1.2 (9)
O4^i^—Mn—N1—C12	−123.4 (4)	C10—C9—C8—N1	−0.6 (9)
Mn—O1—C1—O2	−31.6 (7)	C11—C10—C9—C8	−0.2 (8)
Mn—O1—C1—C2	146.9 (3)	C12—C11—C10—C9	−0.7 (8)
Mn—N1—C8—C9	−178.9 (4)	C13—C11—C10—C9	−170.7 (5)
C12—N1—C8—C9	2.2 (8)	C10—C11—C12—N1	2.4 (8)
C8—N1—C12—C11	−3.2 (8)	C13—C11—C12—N1	173.1 (5)
Mn—N1—C12—C11	177.9 (4)	O3—C13—N2—C14	−179.1 (6)
C13—N2—C14—C15	−111.0 (9)	C11—C13—N2—C14	−3.4 (10)
C16—N2—C14—C15	72.0 (10)	O3—C13—N2—C16	−2.2 (9)
C13—N2—C16—C17	81.2 (9)	C11—C13—N2—C16	173.5 (5)
C14—N2—C16—C17	−101.6 (9)	O3—C13—C11—C10	114.1 (6)
C3—C2—C7—C6	2.0 (8)	N2—C13—C11—C10	−61.6 (8)
C1—C2—C7—C6	−178.8 (5)	O3—C13—C11—C12	−55.9 (7)
C3—C2—C1—O2	−178.0 (4)	N2—C13—C11—C12	128.3 (6)

Symmetry codes: (i) −*x*, −*y*+2, −*z*.

### Hydrogen-bond geometry (Å, °)

**Table:**

*D*—H···*A*	*D*—H	H···*A*	*D*···*A*	*D*—H···*A*
O4—H41···O2^i^	0.99 (4)	1.71 (5)	2.670 (6)	162 (7)
O4—H42···O3^ii^	0.93 (5)	1.85 (5)	2.766 (6)	168 (7)

Symmetry codes: (i) −*x*, −*y*+2, −*z*; (ii) −*x*−1, −*y*+2, −*z*.
